# Controlling the radical-induced redox chemistry inside a liquid-cell TEM†
†Electronic supplementary information (ESI) available: General model description of the water radiolysis, reaction rates and *G*-values. Estimation of the reaction-rate constants of the water radiolysis products with gold species. Sensitivity analysis of the temperature-dependent part of the H_2_ and O_2_ solubility. Model details for the verification by low-dose experiments using γ radiation. *Ex situ* SAED analysis of the conformation of gold NPs. See DOI: 10.1039/c9sc02227a


**DOI:** 10.1039/c9sc02227a

**Published:** 2019-08-16

**Authors:** Bojan Ambrožič, Anže Prašnikar, Nejc Hodnik, Nina Kostevšek, Blaž Likozar, Kristina Žužek Rožman, Sašo Šturm

**Affiliations:** a Jožef Stefan Institute , Department for Nanostructured Materials , Jamova 39 , Ljubljana , Slovenia . Email: saso.sturm@ijs.si; b Jožef Stefan International Postgraduate School , Jamova 39 , Ljubljana , Slovenia; c National Institute of Chemistry , Department of Catalysis and Chemical Reaction Engineering , Hajdrihova 19 , Ljubljana , Slovenia

## Abstract

With Liquid-Cell Transmission Electron Microscopy (LCTEM) we can observe the kinetic processes taking place in nanoscale materials that are in a solvated environment. However, the beam-driven solvent radiolysis, which results from the microscope's high-energy electron beam, can dramatically influence the dynamics of the system. Recent research suggests that radical-induced redox chemistry can be used to investigate the various redox-driven dynamics for a wide range of functional nanomaterials. In view of this, the interplay between the formation of various highly reactive radiolysis species and the nanomaterials under investigation needs to be quantified in order to formulate new strategies for nanomaterials research. We have developed a comprehensive radiolysis model by using the electron-dose rate, the temperature of the solvent, the H_2_ and O_2_ gas saturation concentrations and the pH values as the key variables. These improved kinetic models make it possible to simulate the material's specific radical-induced redox reactions. As in the case of the Au model system, the kinetic models are presented using Temperature/Dose-rate Redox potential (TDR) diagrams, which indicate the equilibrium [Au^0^]/[Au^+^] concentration ratios that are directly related to the temperature-/dose-rate-dependent precipitation or dissolution regions of the Au nanoparticles. Our radiolysis and radical-induced redox models were successfully verified using previously reported data from low-dose experiments with γ radiation and experimentally *via* TDR-dependent LCTEM. The presented study represents a holistic approach to the radical-induced redox chemistry in LCTEM, including the complex kinetics of the radiolysis species and their influence on the redox chemistry of the materials under investigation, which are represented here by Au nanoparticles.

## Abstract 2

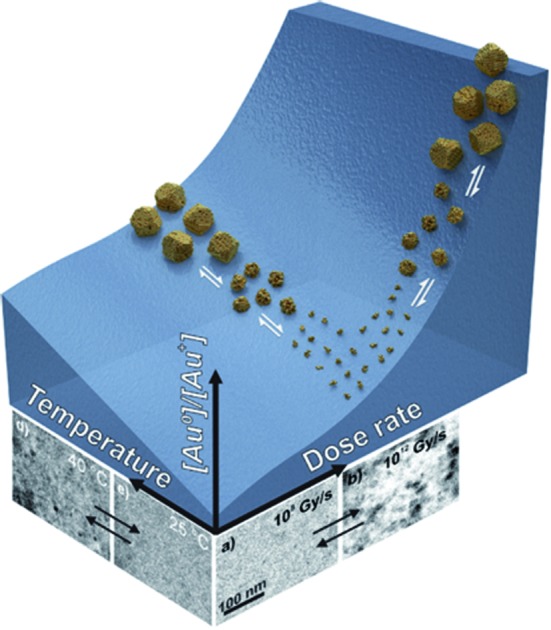
A holistically described radical-induced redox chemistry modelling allows for a direct assessment of the *in situ* experiments inside a liquid-cell TEM.

## Introduction

A transmission electron microscope (TEM) allows the direct imaging and spectroscopy of nanoparticles (NPs). The TEM is conventionally operated in a high vacuum, which requires the samples to be dehydrated. Recent advances have partially overcome this limitation with the use of specialized TEM harnesses, *i.e.*, liquid cells, that can maintain the liquid in a closed environment, allowing the imaging and spectroscopy of samples in liquid media without affecting the vacuum in the microscope.^1^ The samples are held between two silicon chips that have electron-transparent windows made from silicon nitride^2^ or graphene.^3^ This allows us to study dynamic phenomena taking place in the materials in solvated environments^4^ with high spatial and temporal resolution.^5^,^6^

Several previous studies were focused on the precipitation and dissolution dynamics of different nanostructured systems utilizing electron-beam-induced phenomena inside a liquid-cell TEM, for example, Cu and Ni nanocrystals,^7^ Pd dendritic nanostructures,^8^ alloys such as Ag–Pd^7^ or more complex structures like CaCO_3_ (ref. 9) and CeO_2_.^10^ One of the most commonly studied materials using the liquid-cell electron-microscopy (LCTEM) technique is gold,^11^–^14^ with the aim being to investigate the dynamics of the precipitation and dissolution of Au NPs in various aqueous environments. The findings revealed a complex interplay of thermodynamic and kinetic parameters that influence the final morphologies, begging questions about the influence of electron-beam-induced phenomena.^11^,^12^,^15^–^21^

Dynamic LCTEM has tremendous potential when it comes to probing and understanding the underlying mechanisms of nucleation and the early growth stages of nanomaterials.^9^,^16^ On the other hand, there are major challenges related to the interaction effects between the electron beam and the solvent, which can modify the reaction kinetics to such an extent that they can no longer be directly related to the experiments.^21^ It is well documented that these effects are the result of water radiolysis due to the interaction between water and the energy-intense (80–300 keV) electron beam.^22^–^24^ Here, radiolysis is defined as the electron-beam-induced degradation of a water-based electrolyte as a consequence of inelastic scattering.^18^,^25^ There are three separate stages of this radiolysis: the physical stage, which lasts up to 10^–15^ s, the physicochemical stage, which dominates between 10^–15^ and 10^–12^ s, and the chemical stage, taking place for time scales longer than 10^–12^ s. We focused on the chemical stage after 10^–6^ s, where the radiolysis species are considered to be homogeneously distributed. Several types of reactions occur in this stage: reactions between different radiolysis species, reactions between radiolysis species and gases, and reactions between the radiolysis and the Au species.

Fifteen radiolytic species are known to form in water: solvated electrons (e_aq_^–^), hydrogen radicals (H˙), hydroxyl radicals (OH˙), hydrogen (H_2_), hydrogen peroxide (H_2_O_2_), hydroperoxyl radicals (HO_2_˙), hydroperoxyls (HO_2_^–^), protons (H^+^), oxygen (O_2_), superoxides (O_2_˙^–^), oxygen anions (O˙^–^), ozone (O_3_), hydrogen trioxy radicals (HO_3_˙), ozone anions (O_3_˙^–^), and hydroxide (OH^–^). Of these, the most reactive components are the reducing solvated electrons (e_aq_^–^) with a very negative standard redox potential of *E*^0^ = –2.90 V_SHE_ (ref. 26) and the hydroxyl radicals (OH˙) that act as a strong oxidizing agent (*E*^0^ = +1.8 V_SHE_ (ref. 27)). Consequently, the overall chemical reactions between the radiolytic species and the sample during the LCTEM need to be resolved before we can make any reliable interpretation of the experimental data. Schneider *et al.*^11^ were the first to quantify this very complex process inside the liquid cell using a radiolysis model to predict the concentration of the radiolysis species as a function of the dose rate. Although the direct imaging of the radiolysis species is not possible in LCTEM, their presence can be, to some extent, detected by indirect approaches. For example, by quantifying the nucleation and dissolution of Au NPs as a function of the equilibrium concentration ratios of the radiolysis products that act as oxidizing and reducing species,^11^ by using silver NPs as a calibration standard to quantify the effect of the dose rate (as a silver precursor is known to follow a simple, one-electron reduction reaction),^18^ or by observing the formation of a gas bubble inside the liquid cell.^22^

We are proposing a holistic approach that goes well beyond the existing radiolysis models^11^ and will allow us to predict the radical-induced redox chemistry inside a liquid-cell TEM and so design and control the growth of nanostructures. The new kinetic radical-induced redox model considers the following parameters: electron-dose rate, temperature, the formation of H_2_ and O_2_ gaseous phases and the initial pH. Until now, radiolysis models did not include the reactant species with their rate constants and were thus limited to a qualitative assessment of the nanomaterials' precipitation/dissolution kinetics. In our new radiolysis model, where radical-induced redox chemistry is included, the equilibrium concentration of Au ions, [Au^+^], and solid Au, [Au^0^], is included in the model like any other species. The experimental conditions that were used for the gold precipitation and dissolution are in accordance with several previously performed LCTEM investigations,^2^,^6^,^11^,^13^,^28^ allowing us to make a direct comparison. With more detailed knowledge of the radical-induced redox mechanisms, our model offers better control over the experimental conditions in the liquid-cell TEM, which can now be controlled to influence the properties of material systems.

## Results and discussion

### The description of the kinetic radical-induced redox model for gold

This section presents an overview of the calculation for the kinetic radical-induced redox model. The details of the chemical kinetics, the reaction-rate constants, the primary yields and the evaluation of various model parameters, which are experimentally difficult to assess, are included in the ESI.†

The temperature-/dose-rate-dependent kinetic water-radiolysis model consists of reactions between the Au and the radiolytic species, being valid in the temperature range 20–100 °C for acidic and neutral initial solutions. For clarity, the calculated diagrams that are compared with the LCTEM are shown in the same temperature range of 20–60 °C, matching the experimental conditions. The model includes the following species: e_aq_^–^, H˙, OH˙, H_2_, H_2_O_2_, HO_2_˙, HO_2_^–^, H^+^, O_2_, O_2_˙^–^ and OH^–^. Other species (O˙^–^, O_3_, HO_3_˙, O_3_˙) were not included in the model due to their low concentrations at neutral and low initial pH and the lack of reaction-rate constants. The validation of this simplification can be found in the ESI, Section 1.1.† The reaction-rate coefficients were taken from the literature^29^ and rewritten in the shape of the Arrhenius relationship. The primary yields, *i.e.*, the *G*-values of the water radiolysis for the temperature range 20–100 °C were linearly interpolated from the tabulated *G*-values at 20 °C and 100 °C,^29^ respectively. The kinetic constants and more details about the calculation of the kinetic model can be found in the ESI, Section 1.1.†

The kinetic model was further corrected to take into account the presence of the gaseous phase. A common phenomenon observed during LCTEM is the formation of gas bubbles,^22^,^24^,^30^,^31^ implying that the solubility of the formed gases in the liquid is exceeded during LCTEM. This is mainly due to the formation of molecular hydrogen and oxygen during the radiolysis.

For example, by comparing the calculated equilibrium concentrations of H_2_ and O_2_ dissolved in the aqueous solution with their corresponding saturation concentrations at the limiting pressure of 1 bar and in the temperature range 20–60 °C, calculations predict the formation of a gas bubble already at dose rates higher than 10^6^ Gy s^–1^. The effect of window bulging,^34^,^35^ due to the presence of a differential pressure between the liquid in the cell and the vacuum in the TEM column can further decrease the initial cell pressure, which would provoke the gas-bubble formation even at lower dose rates. The lower pressure limit in the liquid-cell TEM is set by the water vapor pressure, which is around 0.02 bar at 20 °C.

**Fig. 1:**
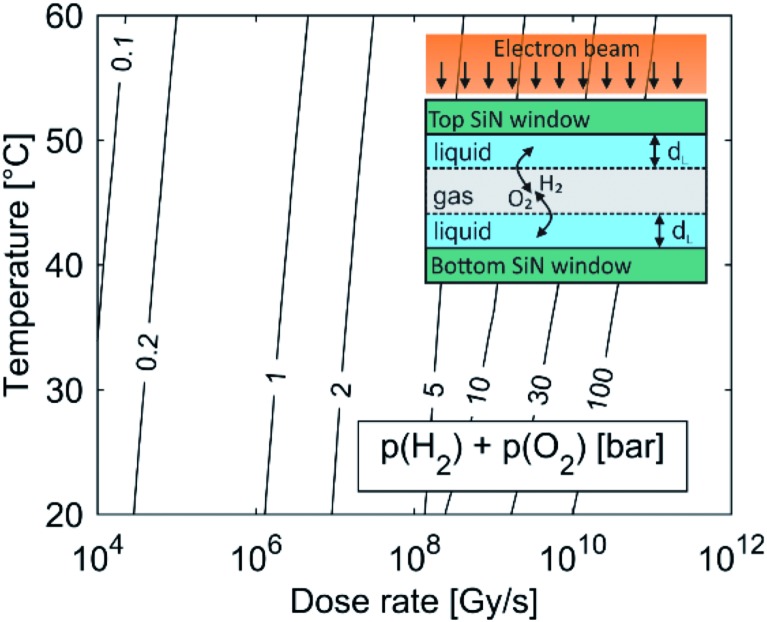
Pressure due to dissolved H_2_ and O_2_ in the equilibrium state for the water-radiolysis simulation. The initial pH is set to 2.8, with no initially dissolved O_2_ or H_2_. The inset shows a schematic representation of the LCTEM system with the gaseous phase. The liquid layer is very thin (*d*_L_ ∼ 50 nm), allowing O_2_ and H_2_ transfer between the liquid and the gas in a period of μs.

Fig. 1 shows the importance of gas-phase formation in the model, from two separate aspects. First, once the gas bubble is formed the equilibrium pressure due to the high concentration of H_2_ and O_2_ in the liquid cell can rise significantly and will continue to increase with an increased dose rate. The effect of a temporal pressure increase at different dose rates is emphasized in the ESI, Section 1.1 (Fig. S2†). Second, the formation of a thin liquid film (*d*_L_ ∼ 50 nm (ref. 22)) in real systems enables fast O_2_ and H_2_ transfer between the liquid and the gas in a period of μs (inset figure). Due to the fast transfer rate between the gas and the liquid phase we assumed that the gas partial pressure and the dissolved O_2_ and H_2_ concentrations in the aqueous solution were in equilibrium. The temperature-dependent solubility values for H_2_ and O_2_ were obtained from NIST^32^,^33^ and the initial pH is set to 2.8, assuming that all the liquid in the cell was evenly irradiated by the electron beam.

As the partial pressure of water only changes from 0.023 bar at 20 °C to 0.199 bar at 60 °C we can assume that the influence of the water's partial pressure on the overall pressure in the gas phase is negligible. This suggests that the pressure increase in the closed cell is predominately controlled by the electron dose rate, which provokes the formation of H_2_ and O_2_ due to water radiolysis. It was experimentally confirmed that a liquid cell with similar dimensions can withstand a pressure of 4 bar.^34^ In practice, a large pressure increase is only expected at high dose rates, since only a small portion of the solution is irradiated by the electron beam. The pressure can also be partially compensated by bowing of the SiN window.^35^

**Fig. 2:**
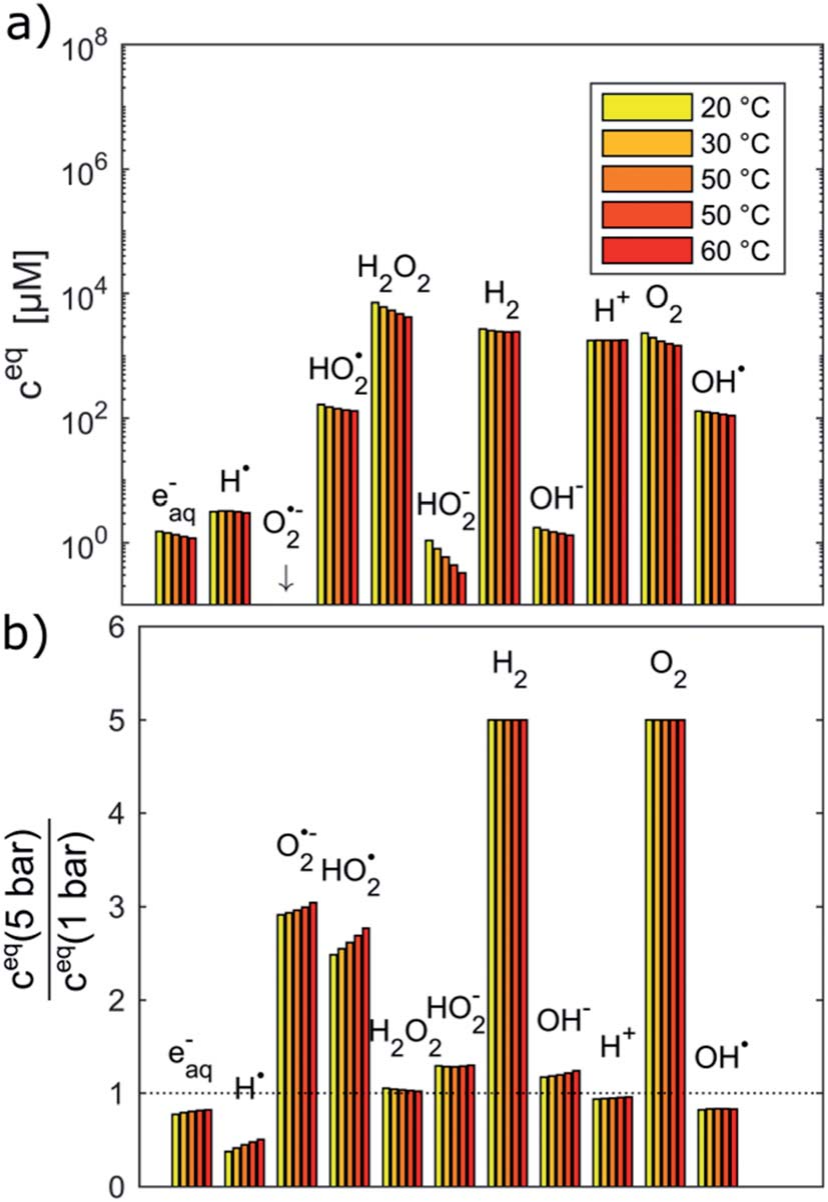
Equilibrium concentrations of radiolysis species in water at a dose rate of 10^7^ Gy s^–1^, pH_initial_ = 2.8 and the temperature range 20–60 °C calculated at (a) 1 bar and (b) relative difference of equilibrium concentrations between 1 and 5 bar.

Fig. 2 shows the equilibrium concentrations of the radiolysis products under typical LCTEM conditions for the Au system: a dose rate of 10^7^ Gy s^–1^ and an initial pH of 2.8 in the temperature range 20–60 °C. The equilibrium concentrations of the radiolysis species were calculated at two limiting pressures in the liquid cells, *i.e.*, at 1 bar and 5 bar (Fig. 2). The pressure-dependent differences in the equilibrium concentration of the radiolysis species are clearly visible (Fig. 2b). The concentrations of H˙, e_aq_^–^ and OH˙ decrease by 20–50% when the pressure increases from 1 bar to 5 bar. Moreover, these graphs also indicate that the temperature influences the equilibrium concentrations of the radiolysis species (notice that the concentrations in Fig. 2a are on a logarithmic scale). Importantly, all of them exhibit the same decreasing trend with the temperature increase.

To estimate the influence of the radiolysis products on the precipitation/dissolution of Au the standard reduction potentials *vs.* standard hydrogen electrode (SHE) of the relevant half-cell reactions are listed in Table 1.

**Table 1:** Standard electrode and Nernst electrode potentials *vs.* SHE of the involved half-cell reactions

Half cell	*E*_0_ [V]	*E*_NERNST_ [V]	Source *E*_0_
e^–^ + [AuCl_2_]^–^ ⇌ Au^0^ + 2Cl^–^	1.15	0.97 ± 0.03	36
e^–^ + H_2_O ⇌ e_aq_^–^	–2.79	–2.28 ± 0.36	26
e^–^ + H^+^ ⇌ H˙	–2.23	–2.09 ± 0.15	26
O_2_ + H^+^ + e^–^ ⇌ HO_2_˙	–0.04	–0.27 ± 0.12	27
2H_2_O + 2e^–^ ⇌ H_2_ + 2OH^–^	–0.83	–0.26 ± 0.42	37
2H^+^ + 2e^–^ ⇌ H_2_	0.00	–0.09 ± 0.05	37
O_2_ + e^–^ ⇌ O_2_˙^–^	–0.33	0.05 ± 0.14	27
O_2_ + 2H^+^ + 2e^–^ ⇌ H_2_O_2_	0.70	0.49 ± 0.12	37
O_2_^–^ + H^+^ + e^–^ ⇌ HO_2_^–^	1.02	0.65 ± 0.16	27
O_2_ + 2H_2_O + 4e^–^ ⇌ 4OH^–^	0.40	0.78 ± 0.28	37
HO_2_˙ + e^–^ ⇌ HO_2_^–^	0.73	0.88 ± 0.24	27
O_2_˙^–^ + 2H^+^ + e^–^ ⇌ H_2_O_2_	1.73	0.92 ± 0.28	27
O_2_ + 2H^+^ + 4e^–^ ⇌ 2H_2_O	1.27	0.98 ± 0.12	37
H_2_O_2_ + 2H^+^ + e^–^ ⇌ 2H_2_O	1.76	1.05 ± 0.25	37
HO_2_˙ + H^+^ + e^–^ ⇌ H_2_O_2_	1.46	1.18 ± 0.13	38
OH˙ + e^–^ ⇌ OH^–^	1.83	1.95 ± 0.12	27

The reductive potentials *vs.* SHE using the Nernst equation for various *in situ* equilibrium conditions that are experimentally more relevant are additionally calculated. The average *E*_NERNST_ was calculated for the following conditions: pH_initial_ 2.8, dose rates 10^6^ to 10^12^ Gy s^–1^, temperature 20–60 °C. From the reductive potentials of the half cells for Au and different radiolysis products, it is clear that the Au species can be reduced in the presence of e_aq_^–^, H˙, H_2_O_2_, HO_2_^–^, H_2_, HO_2_˙, O_2_˙^–^ and can be oxidized in the presence of OH˙ radicals. The strongest reducing agents are e_aq_^–^ (*E*_0_ = –2.79 V_SHE_, *E*_NERNST_ = –2.28 ± 0.36 V_SHE_) and H˙ (*E*_0_ = –2.23 V_SHE_, *E*_NERNST_ = –2.09 ± 0.15 V_SHE_), which cause rapid metal precipitation,^39^ while OH˙ (*E*_0_ = 1.83 V_SHE_, *E*_NERNST_ = 1.95 ± 0.12 V_SHE_), as a strong oxidative agent, causes metal dissolution.^12^ The reaction-rate coefficients between the radiolysis products and the Au species indicate that the H˙, OH˙ radicals and e_aq_^–^ have a much stronger effect on the gold species compared to the other solutes (Table 2). More details about the interaction between the radiolysis products and the gold species in the liquid-cell TEM can be found in the ESI, Section 1.2.†

**Table 2:** Reaction-rate constants of radiolysis products with gold species

Au spec.	Radiolysis spec.	*k*_20°C_ [M^–1^ s^–1^]	*E*_A_ [kJ mol^–1^]	*A*_calculated_
Au^+^	e_aq_^–^	8.0 × 10^9^	12.98	1.64 × 10^12^
	H˙	8.0 × 10^9^	15.09	3.91 × 10^12^
	H_2_O_2_	0	0	0
	HO_2_^–^	1.89	25.8	7.46 × 10^4^
	H_2_	7.4 × 10^–3^	94.1	4.28 × 10^14^
	HO_2_˙	1.89	25.8	7.46 × 10^4^
	O_2_˙^–^	1.89	25.8	7.46 × 10^4^
Au^0^	OH˙	1.83 × 10^9^	13.0	3.80 × 10^11^

Further explanation of the reaction rates can be found in the ESI, Table S3.†

The primary aim of this study is to model and control the radical-induced redox chemistry in a liquid-cell TEM that is based on the precipitation/dissolution of Au NPs, which we used as a model system. Accordingly, we have simplified the model to a homogeneous system, which means the reduced Au species are introduced in the form of concentration and are all available for oxidation reactions. Additionally, it is assumed that all the Au ions are in the form of Au^+^, as we want to observe only the transition between the Au^0^ atoms and the Au^+^ ions. The concentrations of Au species are incorporated into the model like any other component, where the reaction-rate constants between the radiolysis products and the Au species were taken from Table 2. The corresponding rate of Au-species production is shown in eqn (1):1
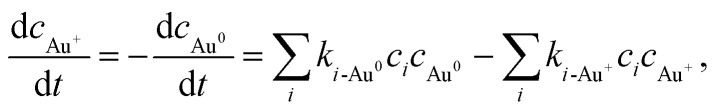
where *c*_Au^+^_ and *c*_Au^0^_ represent the concentrations of gold ions and gold atoms, respectively. The reaction-rate constants for the reactions between the *i* compound with the gold species are indicated by *k*_*i*-Au^0^_ and *k*_*i*-Au^+^_. Using this equation, we estimated that the steady-state, *i.e.*, the equilibrium, concentration of the gold species is achieved after 10^–3^ s.

The resulting equilibrium ratio of [Au^0^]/[Au^+^] in our model can be taken as an indicator of the reductive/oxidative ratio between the ionic, *i.e.*, dissolved, and the solid, *i.e.*, precipitated, gold. The latter will form in the liquid cell as Au NPs. These theoretically obtained values can be translated into the LCTEM experiment by assuming that the Au precipitation or dissolution is directly related to the equilibrium ratio [Au^0^]/[Au^+^], thus providing the same kind of information. For example, a [Au^0^]/[Au^+^] gradient increase indicates the gold's tendency for precipitation and growth, resulting in the formation of Au NPs. In contrast, a [Au^0^]/[Au^+^] gradient decrease signifies either the inability of Au to precipitate or the dissolution of the already-formed Au NPs. These are the phenomena that can be easily observed during LCTEM.

**Fig. 3:**
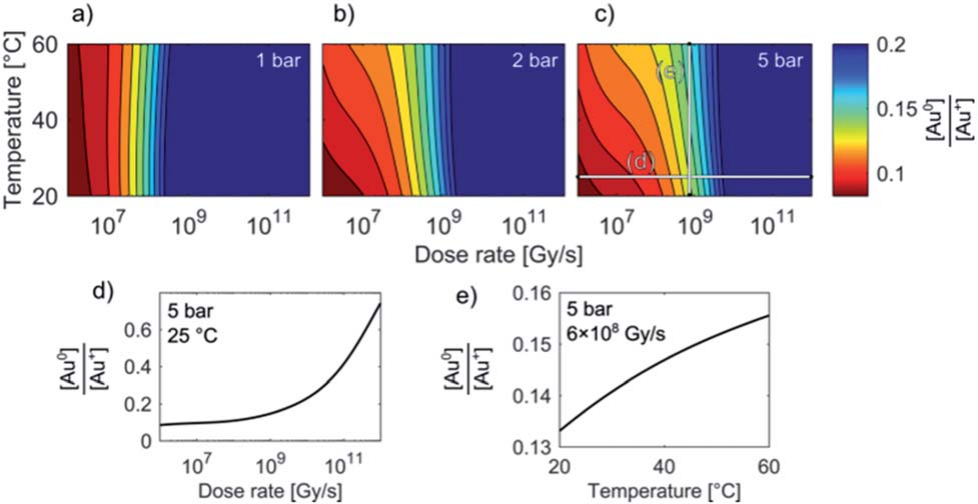
TDR diagrams (a–c). Note that the stability regions for the equilibrium [Au^0^]/[Au^+^] concentrations in the liquid-cell TEM are, in addition to dose-rate and temperature, also pressure dependent. Initial [Au^+^] = 1.5 mM, pH_initial_ = 2.8. (d) and (e) show sections of the Au redox ratio trends from diagram (c).

Temperature/Dose-rate Redox ratio (TDR) diagrams, showing different redox [Au^0^]/[Au^+^] stability regions, are shown in Fig. 3. The TDR diagrams are calculated for the dose-rate range 10^6^ to 10^12^ Gy s^–1^, temperature range 20–60 °C, and at the H_2_ + O_2_ pressures 1 bar, 3 bar, and 5 bar, with the molar ratio in the gaseous phase H_2_/O_2_ = 2. The resulting equilibrium redox concentration ratio of [Au^0^]/[Au^+^] was obtained for an initial concentration of Au^+^ species of 1.5 mM and a corresponding pH of 2.8. The calculated TDR diagrams are characterized with distinctive regions where the conditions in the liquid-cell TEM tend towards a reducing environment that will promote the precipitation of Au NPs (blue). In contrast, the oxidative environment (dark red) will either prevent the formation of Au NPs or promote the Au NPs' dissolution. The following “rainbow” color code indicates the intermediate conditions between the oxidative and reductive extremes.

When the pressure in the cell is set to 1 bar the temperature does not have any significant effect on the Au redox ratio (Fig. 3). In that case, a change in the dose rate has a much larger impact on the Au redox ratio when compared with a temperature change. This implies that the precipitation of gold is positively and monotonously correlated with an increase in the dose rate, as was previously reported by Schneider *et al.*^11^

However, our results indicate that even at intermediate dose rates the saturation concentration for the solubility of H_2_ and O_2_ in the aqueous solutions will be exceeded and the radiolysis model is better defined by considering the elevated-pressure regimes inside the LCTEM system. The resulting model at elevated pressure (5 bar) indicates that the effect of temperature on the Au redox ratio starts to become significant, which is most pronounced at the low and intermediate dose rates that are typically used during LCTEM. The effect of the temperature on the redox conditions can be explained by the scavenging effect of the H_2_ and O_2_ species, *i.e.*, H_2_ is a strong OH˙ scavenger, while O_2_ is a strong scavenger of H˙ and e_aq_^–^. Due to the presence of the gaseous phase, the concentrations of H_2_ and O_2_ are mainly dependent on the finite pressure and solubility limits. Moreover, with a temperature increase, the O_2_ solubility will decrease at higher rates when compared to the H_2_ solubility. As a result, a smaller amount of H˙ and e_aq_^–^ scavengers at higher temperatures pushes the system toward more reductive conditions, as is evident in Fig. 3 for pressures of 3–5 bar. The sensitivity analysis of the temperature-dependent part of the H_2_ and O_2_ solubility supports this explanation and is elaborated in the ESI, Section 1.3.†

In short, the change of the reaction-rate constants between the gold and the radiolysis species due to a temperature variation between 20 °C and 60 °C does not directly influence the gold redox ratio. The temperature change influences the maximum concentration of O_2_ and H_2_ in the liquid phase due to the thermodynamic solubility of these two gases. This effect is even more pronounced at higher pressures, as observed in Fig. 3a–c.

### Model verification by low-dose experiments using γ radiation

**Fig. 4:**
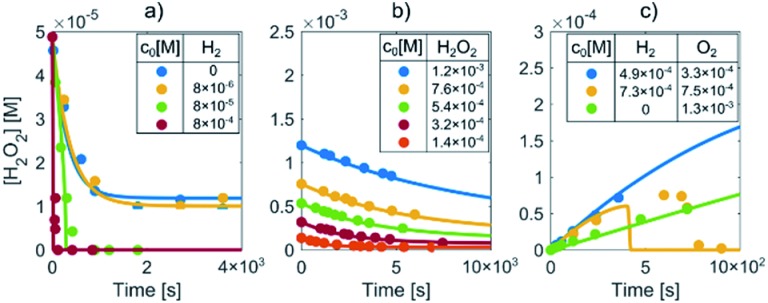
Determination of H_2_O_2_ concentrations during the irradiation of water with low-dose γ radiation for different initial (a) H_2_, (b) H_2_O_2_ and (c) H_2_ and O_2_ concentrations (marked by color-coded dots). The corresponding color-coded solid lines represent the prediction of our radiolysis model.

Prior to the LCTEM, the model was validated with a quantitative comparison using literature reports^40^–^42^ of radiolysis products obtained by irradiating water with low-dose γ radiation. Electron radiation is a low-LET (linear energy transfer) radiation and is similar to γ radiation. Hill^43^ calculated the primary yields for different electron energies and observed constant yields above 10 keV. Temperature-dependent primary yields were obtained for water at neutral pH from Elliot and Bartels.^29^ The experiments were performed at different dose rates and solute concentrations. Fig. 4 shows different sets of experimental H_2_O_2_ concentration data obtained by irradiating water with low-dose γ radiation under various experimental conditions as a function of time. It is worth mentioning that every dataset is obtained from a different literature source.^40^–^42^ Only the experimental data for concentration variations of H_2_O_2_, H_2_ and O_2_ are reported here. More details about the model's verification can be found in the ESI, Table S6.† Individual experimental data points are represented by color-coded dots, while the corresponding color-coded solid lines are obtained from the kinetic radiolysis model developed in this study using the given low-dose γ-radiation experimental conditions. For example, Fig. 4a shows the concentration variations of H_2_O_2_ for different initial concentrations of H_2_, while Fig. 4b shows H_2_O_2_ variations for different initial values of H_2_O_2_. Fig. 4c indicates the concentration changes of H_2_O_2_ when varying the initial concentration of both H_2_ and O_2_. Remarkably, although not at exactly the same time, our model accurately predicts the abrupt drop of the H_2_O_2_ concentration for the case of initial H_2_ and O_2_ concentrations of 7.31 × 10^–4^ and 7.50 × 10^–5^ M, respectively (shown in the yellow-coded color). By comparing the result of our kinetic radiolysis model with the experimental data for low-dose γ radiation (∼1 Gy s^–1^),^40^–^42^ it is evident that the proposed radiolysis model fits very well with the experimentally obtained data.

### Model verification based on Au precipitation/dissolution liquid-cell transmission electron microscopy (LCTEM)

**Fig. 5:**
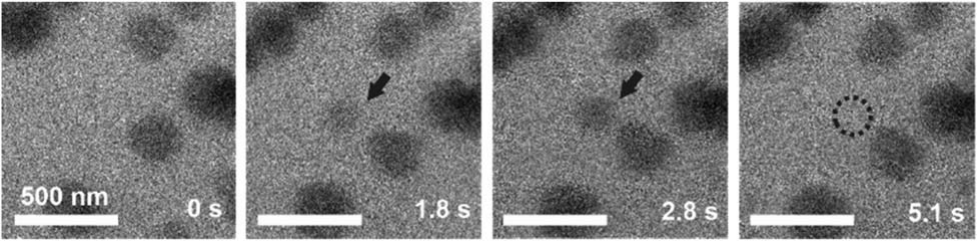
TEM micrographs indicating the precipitation and dissolution of Au NPs at a constant dose rate of 10^9^ Gy s^–1^ and a temperature of 20 °C. The encircled areas and arrows indicate the sequence of precipitation and dissolution for a single Au NP under equilibrium conditions.

LCTEM was used to compare the observed Au NPs' precipitation, growth and dissolution dynamics with the calculated equilibrium Au redox ratio, [Au^0^]/[Au^+^]. First, the equilibrium conditions where the Au NPs were growing and dissolving at approximately the same rates were experimentally determined. Fig. 5 shows the time sequence of the precipitation and dissolution dynamics of the Au NPs at a constant dose rate of 10^9^ Gy s^–1^ and at a temperature of 20 °C. This absorbed dose rate was a result of a constant electron-beam exposure of approximately 200 e^–^ Å^–2^ s^–1^, assuming the 200 keV electron stopping power in water (S) equal to 2.78 MeV cm^2^ g^–1^ and a density of 1 g cm^–3^. A representative region where the Au NPs exhibit precipitation, growth and dissolution on a time scale of 5 s is marked by an arrow or dashed-line circle. At 0 s, the Au NPs are not yet formed; they appear at 1.8 s and remain stable for approximately 3 s, followed by complete dissolution at 5.1 s. The *ex situ* SAED analyses confirmed that the NPs are face-centered-cubic gold (ESI, Section 3†).

**Fig. 6:**
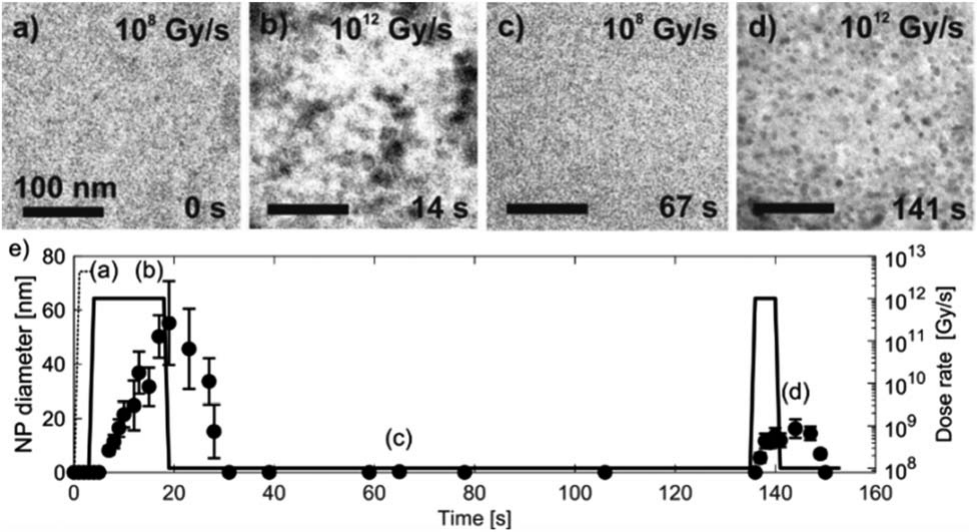
Time-sequenced TEM micrographs acquired from the same specimen area at 25 °C and alternating electron-dose rates (a) 10^8^, (b) 10^12^, (c) 10^8^ and (d) 10^12^ Gy s^–1^. (e) The corresponding graph of the Au NPs' precipitation and dissolution. The data points indicating the average NP diameters were determined from 10 individual NPs measured at a specific time.

Knowing the experimentally determined [Au^0^]/[Au^+^] equilibrium conditions for this set of experiments, the proposed radiolysis model for radical-induced redox chemistry in a liquid-cell TEM by varying the electron dose rate at ambient temperature was further verified. The results of the experiment are shown in Fig. 6. The experimental dose rate was set to 10^8^ Gy s^–1^, corresponding to an electron-beam exposure of around 20 e^–^ Å^–2^ s^–1^ for 4 s (Fig. 6a), just below the expected dose rate for the precipitation of Au NPs, as determined in the previous experiment. Accordingly, at a dose rate of 10^8^ Gy s^–1^ no NPs were formed. Then, after 4 s the dose rate was increased from 10^8^ Gy s^–1^ to 10^12^ Gy s^–1^ (beam exposure of around 2 × 10^5^ e^–^ Å^–2^ s^–1^) for 10 s, which initiated the precipitation of Au NPs and their growth to an average size of 55 nm (Fig. 6b). The dose rate was then quickly reduced back to the initial 10^8^ Gy s^–1^, whereupon the Au NPs started to shrink, and in less than 10 s the NPs were completely dissolved (Fig. 6c). The experiment was repeated in the same area 110 s later to confirm that the phenomenon is repeatable. In this repeat experiment the area was exposed to a dose rate of 10^12^ Gy s^–1^ for 5 s, instead of the 15 s in the previous experiment. The shorter exposure time resulted in the formation of NPs with an average size of 16 nm (Fig. 6d). When the dose rate was decreased for the second time to 10^8^ Gy s^–1^ the NPs dissolved in approximately 10 s. A quantitative evaluation of the whole experiment is presented in Fig. 6e. It shows the variations in the size of the Au NPs as a function of the electron dose rate at room temperature. The line in the image represents the selected dose rate, while the circles indicate the average sizes of the NPs. These results confirmed that the electron-dose-dependent LCTEM is reversible and correlates with the predictions of the TDR diagrams, where the Au NPs' precipitation or dissolution at an ambient temperature is positively correlated with the variations in the Au redox concentration ratio, *i.e.*, [Au^0^]/[Au^+^] as a function of the electron dose rate. Similar precipitation/dissolution trends were observed in studies by Schneider *et al.*^11^ and Ahn *et al.*^16^

**Fig. 7:**
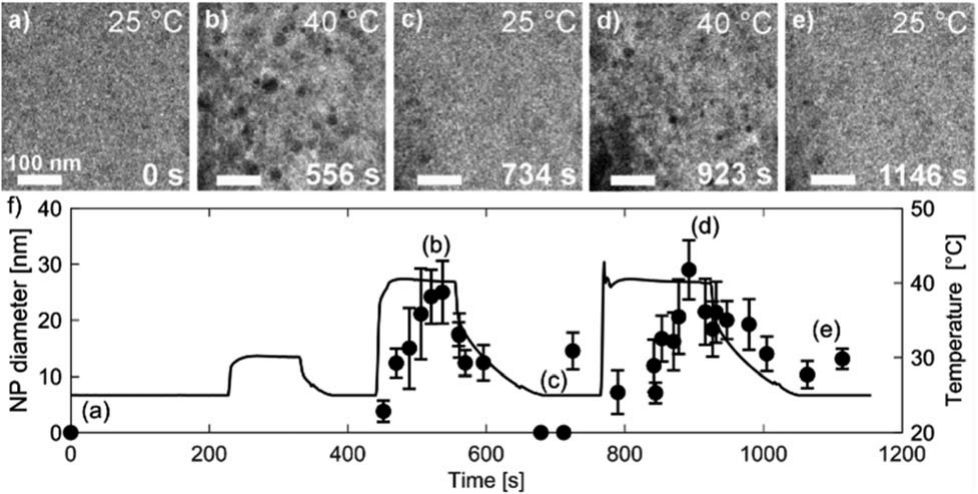
Time-sequenced TEM micrographs acquired from the same specimen area at a fixed electron-dose rate of 6 × 10^8^ Gy s^–1^ and alternating temperatures of (a) 25 °C, (b) 40 °C, (c) 25 °C, (d) 40 °C and (e) 25 °C. (f) Corresponding graph of the Au NPs' precipitation and dissolution. The temperature–time profile is shown as a black line. The data points indicating the average NP diameters came from 10 NPs measured at a specific time.

Fig. 7 shows TEM micrographs of the precipitation/dissolution of the Au NPs as a function of temperature between 25 °C and 40 °C over a period of 1200 s. The electron dose rate was fixed at 6 × 10^8^ Gy s^–1^, at an initial temperature of 25 °C, just below the experimentally determined boundary conditions for the Au NPs' precipitation. The temperature-dependent experiment started at 25 °C, with the same specimen area being observed for 215 s (Fig. 7a), which is several orders of magnitude longer than the time needed to reach the equilibrium concentration of radiolysis species. This experiment demonstrates that the Au-based aqueous solution is stable at electron-dose rates of around 10^8^ Gy s^–1^ at 25 °C. Then, after a relatively long exposure time, the temperature was increased to 30 °C for a period of 110 s. These conditions did not result in any precipitation of Au NPs, in accordance with the relatively broad [Au^0^]/[Au^+^] gradient field in the temperature domain, as indicated in the TDR diagrams for elevated pressures. Next, the temperature was increased to 40 °C for Δ*t* = 110 s, and the Au NPs started to form with an average diameter of approximately 25 nm (Fig. 7b). Then, when the temperature was decreased to 25 °C these precipitated Au NPs were completely dissolved in approximately 100 s (Fig. 7c).

The reversibility of the phenomenon was checked by repeating the temperature changes. First, the temperature was set to 40 °C for Δ*t* = 160 s, which resulted in the precipitation of Au NPs with an average diameter of approximately 30 nm (Fig. 7d), after which the temperature was reduced to 25 °C and the previously precipitated Au NPs were again completely dissolved (Fig. 7e). In this way we were able to confirm the temperature-dependent reversibility of the Au precipitation–dissolution mechanism. The corresponding graph of the temperature-dependent Au NPs' precipitation and dissolution behavior in the TEM images is shown in Fig. 7f.

These observations are in contrast to what we would expect to see in a conventional experiment, *i.e.*, neglecting the radiolysis-induced redox chemistry, where the precipitation of Au NPs from an aqueous solution at low pH values is provoked by elevated temperature.^44^,^45^ Furthermore, such a process is not reversible and cannot lead to Au NPs dissolving as the temperature drops. This phenomenon can only be explained in terms of the temperature changes modifying the equilibrium concentration of the radiolysis species, thereby changing the Au redox concentration ratios, finally resulting in the reversible precipitation/dissolution of Au NPs. These results are therefore consistent with the [Au^0^]/[Au^+^] trends indicated in the TDR diagrams for elevated pressures (Fig. 3c).

## Experimental section

The extensive simulation algorithms for the radiolysis inside the liquid-cell TEM were built in the MATLAB software platform (MATLAB 2016b, The MathWorks, Inc., Natick, Massachusetts, United States).

An aerated 1.5 mM water solution of gold(iii) chloride trihydrate (chloroauric acid) as a source of gold was used (Sigma Aldrich, >99.9% trace-metal basis). The solution was prepared a few minutes before the start of the experiments to avoid any potential spontaneous Au NP formation. Different sets of LCTEM experiments were performed at various electron-dose rates and temperatures, focused on precipitation and dissolution of the Au NPs.

The TEM (Jeol JEM 2100 TEM) operated at a 200 kV accelerating voltage had a Protochips Poseidon liquid holder with a heating capability (Protochips Inc., Raleigh, NC). Experimental images and videos were recorded using an Orius Model 832 SC1000 CCD camera at 30 frames per second and a theoretical pixel resolution of 0.5 nm. The TEM observation mode was selected to achieve a uniform and parallel illumination of the analyzed area with the electron beam. The electron-dose rate for given electron-optical TEM parameters was calculated according to the expression developed by Schneider *et al.*^11^ (ESI, eqn S(3)†). The electron density for the specimen area, an important parameter for determining the electron-dose rate for a thin liquid layer, was controlled by various combinations between initial gun current, condenser lens and condenser aperture settings in the TEM. The aqueous solution inside the holder was confined in a closed cell between the two silicon chips (referred to as the top and bottom chips). Imaging was performed on the exit side of the bottom chip, in the corner areas of two SiN crossed windows, separated by 50 nm-thick spacers, resulting in a relatively controlled water-layer thickness with minimized window bulging in the area of observation and relatively high theoretical spatial resolution in the sub-nm range.^23^ The top chip contains tungsten heating coils for the external heating of the liquid. Special care was taken in order to prevent any dust entering the chips.^2^

The temperature of the aqueous solutions in the liquid cell was regulated indirectly by applying an electric current through the tungsten coils located on the top chip. The electrical current was applied with a Keithley controller *via* Protochips Poseidon V2.0.4 software (Protochips Inc., Raleigh, NC) with a calibration curve that was provided by the manufacturer. With this system, a temperature between 20 °C and 100 °C could be achieved. In this study the heating rate was 5 °C s^–1^, with the maximum temperature set to 60 °C. The temperature increase of the liquid as a result of the electron-beam irradiation was not taken into consideration, as it was shown in several studies that for the given experimental beam currents the temperature rise is restricted to within a few degrees centigrade.^17^,^22^ The fastest cooling rate from the final temperature (≈60 °C) was estimated to be around 0.6 °C s^–1^. The morphology, composition and crystal structure of the precipitates that were formed during the *in situ* LCTEM were subsequently analyzed in a dried state using a conventional TEM, for example, the selected-area electron diffraction (SAED) analysis and energy-dispersive X-ray spectroscopy (EDXS).

## Conclusions

The proposed TDR diagrams obtained from the improved kinetic radiolysis model provide us with a better simulation of the precipitation, growth and dissolution of Au NPs under a variety of LCTEM conditions. In the LCTEM system the Au redox potential is investigated as a parameter of the electron-dose rate, temperature and pressure. Our new model was verified using experimental literature data obtained from low-dose experiments using γ radiation. The validity of the radiolysis model was then confirmed by LCTEM experiments that were consistent with the TDR diagrams. It was confirmed that the Au NPs precipitate firstly as a result of an increased dose rate and, secondly, due to the combined effect of the increased temperature, pressure and saturation concentration of H_2_ and O_2_, which in both cases shift the equilibrium [Au^0^]/[Au^+^] concentrations in favor of zero-valence gold (Au^0^). Both proposed mechanisms were found to be completely reversible, which suggests that the Au precipitation/dissolution reactions can be fine tuned by altering the parameters inside the liquid-cell TEM.

Modeling the complex redox chemistry inside the liquid-cell TEM allows a real-time nanoscale visualization and manipulation of nanostructured systems in liquid environments. As such, our radiolysis model can, in principle, be implemented in many different branches of nanotechnology and energy-related processes by including appropriate, system-specific, radical-induced redox chemistry.

## Conflicts of interest

There are no conflicts to declare.

## Supplementary Material

Supplementary information
