# Supplementary material for: Controlling the radical-induced redox chemistry inside a liquid-cell TEM
Source: Chem Sci. 2019 Aug 16;10(38):8735–43. doi: 10.1039/c9sc02227a (PMC6991189; doi:10.1039/c9sc02227a)
Supplement: Supplementary file 1 [file SC-010-C9SC02227A-s001.pdf]

Electronic Supplementary Material (ESI) for Chemical Science.
This journal is © The Royal Society of Chemistry 2019

Supporting information:

Received 00th January 20xx,
Accepted 00th January 20xx

DOI: 10.1039/x0xx00000x

Controlling the Radical-Induced Redox Chemistry Inside a Liquid-Cell TEM

Bojan Ambrožič[a,b], Anže Prašnikar[b,c], Nejc Hodnik[c], Nina Kostevšek[a], Blaž Likozar[c], Kristina Žužek Rožman[a], Sašo Šturm[a]

1. DESCRIPTION OF THE KINETIC RADICAL-INDUCED REDOX MODEL FOR GOLD

1.1 General model description of water radiolysis:

The $O^{\bullet-}$, O_3, $HO_3^{\bullet}$, $O_3^{\bullet}$ species were not included in the model due to the lack of any corresponding kinetic data at higher temperatures. These species are predominantly formed in basic solutions. This is due to the deprotonation of the hydroxyl radical ($OH^{\bullet}$) into $O^{\bullet-}$ ($pK_a \approx 11$[1]) and H_2O_2 into HO_2^- ($pK_a \approx 11$[1]). The presence of the oxygen anion and the hydroperoxyl anion is responsible for the formation of the ozone anion and subsequently other ozonide species.[1] To justify the model's simplification, we calculated the ratio between the sum of the equilibrium concentrations of $O^{\bullet-}$, O_3, $HO_3^{\bullet}$, O_3^- and the equilibrium concentrations of the $OH^{\bullet}$ radical, using the correlations for equilibrium concentrations in a homogenous solution [2] at an initial *pH* 7 and 20 °C (Fig. S1). At 10^{10} Gy/s (upper limit of the power-law model) the ratio is 0.012; therefore, there are approximately 80-times more $OH^{\bullet}$ radicals than other strong oxidizing species. Due to the lower deprotonation rate at lower pH, the ratio of these concentrations is even lower, leaving the $OH^{\bullet}$ radical as the main strong oxidizing agent.

[a.] *Jožef Stefan Institute, Department for Nanostructured Materials, Jamova 39, Ljubljana, Slovenia.*
[b.] Jožef Stefan International Postgraduate School, Jamova 39, Ljubljana, Slovenia.
[c.] *National Institute of Chemistry, Department of Catalysis and Chemical Reaction Engineering, Hajdrihova 19, Ljubljana, Slovenia.*
† Footnotes relating to the title and/or authors should appear here.
Electronic Supplementary Information (ESI) available: [details of any supplementary information available should be included here]. See DOI: 10.1039/x0xx00000x

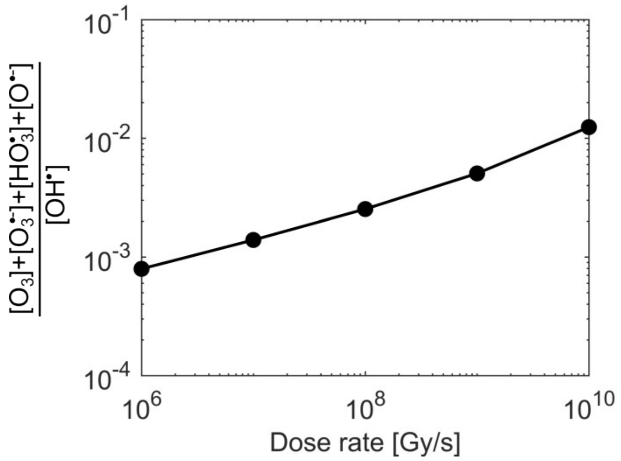

Figure S1. Validity of the simplification of the reaction scheme: the dose-rate dependence of the ratio of the equilibrium concentration between $O^{\bullet-}$, O_3, HO_3, O_3^- and $OH^{\bullet}$ radicals at an initial *pH* = 7 and 20 °C.

According to the Arrhenius plots in Elliot *et al.*[1], all the reaction-rate constants linearly follow the relation $\ln(k) \propto T^{-1}$ below 100 °C. Therefore, the temperature dependence is written in the form of activation energies and pre-exponential factors (Table S1). Processes in the irradiated matter can be described by a mass-balance equation with the reaction parts and the radiolysis primary yields:

$$\frac{dc_i}{dt} = -\sum_j k_{ij}c_ic_j + \sum_{j,k\neq i} k_{jk}c_jc_k + R_i \quad , \qquad \text{(S1)}$$

where c_i represents the concentrations of the individual species, k_{ij} represents the reaction-rate constant and R_i represent the radiolytic yield. The first part of the right-hand side of the equation describes the consumption rate of the i compound, while the second part describes the formation of the i compound. The radiolytic yield R_i (Eq. S2) is the formation or consumption rate of the i compound by radiolysis.

$$R_i = \frac{\rho\psi G_i}{F}\left[\frac{M}{s}\right] \qquad \text{(S2)}$$

In the above Eq. S2, ρ represents the solvent density, F is the Faraday constant, ψ is the dose rate of the radiation and G_i is a G value, which represents the primary yield of the i compound in the first µs from the start of the radiation. This value is given as the number of molecules created or destroyed per 100 eV of energy deposited. The G values depend on the type of radiation, the media exposed to the radiation and the temperature. They are given in Table S2. The temperature dependence of the G values is calculated using linear interpolation. The dose rate ψ is calculated from Eq. S3.

$$\psi = \frac{S\,10^5 I}{\pi a^2}\left[\frac{Gy}{s}\right]. \qquad \text{(S3)}$$

where S [MeV cm²/g electron] is the density-normalized, stopping power in the medium, I is the beam current and a is the beam radius.

Table S1. Reaction-rate constants for the temperature range between 20 °C and 100 °C[1]

**reagents**	**products**	***A* [*]**	**E_A [kJ mol^{-1} K^{-1}]**
$H^+ + OH^-$	H_2O_2	1.88×10^{13}	12.62
H_2O	$H^+ + OH^-$	1.70×10^{6}	62.37
H_2O_2	$H^+ + HO_2^-$	4.12×10^{6}	43.77
$H^+ + HO_2^-$	H_2O_2	5.59×10^{12}	11.73
$H_2O_2 + OH^-$	$HO_2^- + H_2O$	3.66×10^{12}	13.98
$HO_2^- + H_2O$	$H_2O_2 + OH^-$	4.54×10^{11}	31.74
$e^-_{aq} + H_2O$	$H^\bullet + OH^-$	5.58×10^{6}	31.73
$H^\bullet + OH^-$	$e^-_{aq} + H_2O$	8.52×10^{13}	37.36
$H^\bullet$	$H^+ + e^-_{aq}$	2.84×10^{12}	66.66
$H^+ + e^-_{aq}$	$H^\bullet$	1.98×10^{12}	11.17
$HO_2^\bullet$	$O_2^{\bullet-} + H^+$	2.63×10^{8}	14.58
$O_2^{\bullet-} + H^+$	$HO_2^\bullet$	5.59×10^{12}	11.73
$HO_2^\bullet + OH^-$	$O_2^{\bullet-} + H_2O$	7.13×10^{9}	60.93
$O_2^{\bullet-} + H_2O$	$HO_2^\bullet + OH^-$	3.66×10^{12}	13.98
$e^-_{aq} + OH^\bullet$	OH^-	2.64×10^{12}	10.65
$e^-_{aq} + H_2O_2$	$OH^\bullet + OH^-$	7.75×10^{12}	15.72
$e^-_{aq} + H_2O + O_2^{\bullet-}$	$HO_2^- + OH^-$	4.43×10^{10}	12.98
$e^-_{aq} + HO_2^\bullet$	HO_2^-	2.45×10^{12}	12.98
$e^-_{aq} + O_2$	$O_2^{\bullet-}$	2.53×10^{12}	11.66
$e^-_{aq} + e^-_{aq} + H_2O + H_2O$	$H_2 + OH^- + OH^-$	1.01×10^{10}	20.74
$e^-_{aq} + H^\bullet + H_2O$	$H_2 + OH^-$	2.06×10^{11}	14.93
$H^\bullet + H_2O$	$H_2 + OH^\bullet$	7.39×10^{12}	98.24
$H^\bullet + H^\bullet$	H_2	2.69×10^{12}	15.51
$H^\bullet + OH^\bullet$	H_2O	4.19×10^{11}	9.03
$H^\bullet + H_2O_2$	$OH^\bullet + H_2O$	1.76×10^{11}	21.01
$H^\bullet + O_2$	$HO_2^\bullet$	9.01×10^{11}	10.52
$H^\bullet + HO_2^\bullet$	H_2O_2	5.05×10^{12}	15.09
$H^\bullet + O_2^{\bullet-}$	HO_2^-	5.05×10^{12}	15.09
$OH^\bullet + OH^\bullet$	H_2O_2	9.78×10^{10}	7.48
$OH^\bullet + HO_2^\bullet$	$O_2 + H_2O$	1.31×10^{11}	6.68
$OH^\bullet + O_2^{\bullet-}$	$OH^- + O_2$	8.75×10^{11}	10.84
$H_2 + OH^\bullet$	$H^\bullet + H_2O$	6.55×10^{10}	18.45
$OH^\bullet + H_2O_2$	$HO_2^\bullet + H_2O$	7.72×10^{9}	13.82
$OH^\bullet + HO_2^-$	$HO_2^\bullet + OH^-$	1.00×10^{12}	11.92
$HO_2^\bullet + O_2^{\bullet-}$	$HO_2^- + O_2$	2.62×10^{9}	8.09
$HO_2^\bullet + HO_2^\bullet$	$O_2 + H_2O_2$	2.77×10^{9}	20.07

*for the reactions with one molecule the unit is [s^{-1}], for two molecules [$M^{-1}s^{-1}$], for three molecules [$M^{-2}s^{-1}$] and for four molecules [$M^{-3}s^{-1}$]. We used 55.56 M as the molar concentration of water.

This journal is © The Royal Society of Chemistry 20xx

Table S2. Primary yields, G values, for the temperature range between 20 °C and 100 °C

species	$G_{i\text{-}20}$ °C [N_i/100eV]	$G_{i\text{-}100}$ °C [N_i/100eV]	Source
e^-_{aq}	2.73	3.10	[1]
H^+	3.19	3.61	[a]
OH^-	0.46	0.51	[a]
H_2O_2	0.72	0.59	[1]
$H^•$	0.60	0.71	[1]
$OH^•$	2.75	3.57	[1]
H_2	0.43	0.47	[1]
H_2O	-4.65	-5.26	[b]

[a] H^+ primary yield is calculated using the ratio between $G(H^+)$ and $G(e^-_{aq})$ obtained from experimental measurements [3,4] that are summed in the work of Hill and Smith [5]. Using this ratio the charge-balance yield $G(OH^-)$ to 0.46 at 25 °C and 0.51 at 100 °C, which is a similar value to $G(OH^-)$ = 0.50 for low LET radiation.[6]

[b] Mass-balance calculation.

Temporal pressure increase: A dynamic pressure increase due to radiolysis was estimated by assuming the already-formed gas bubble, composed of H_2 and O_2 gas, exposed specimen area using an electron beam with a diameter of 1 µm and the overall liquid-layer thickness of 100 nm. A constant 10-time cell volume expansion was assumed due to the gas formation, which is in accordance with the prediction of Park et al.[7]. Figure S2 below shows an estimated dynamic pressure increase.

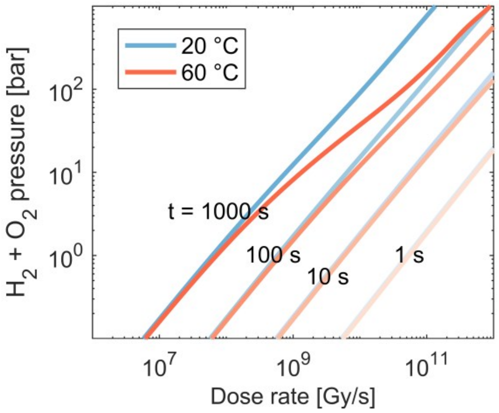

Figure S2. The effect of dynamic pressure increase at different dose rates as a function of time for two limiting temperatures, 20°C and 60°C.

1.2 The interaction between the radiolysis products and the gold species in the liquid-cell transmission electron microscopy (LCTEM) system:

Influence of chloride ions: The chloride is an $OH^•$ scavenger. The main reaction product due to the presence of chloride is $ClOH^{•,-}$[8] which is also a very strong oxidizer ($E_0(ClOH^{•-}/(Cl^-+OH^-))$ = 1.91 V_{SHE}[9]) and can also oxidize Au particles. Due to the large difference in the standard electrochemical potentials between $ClOH^{•-}$ and Au^0, it is assumed that the rate of Au oxidation with $OH^•$ and $ClOH^{•-}$ is similar, which is approximated to a negligible influence on the trends of the Au oxidation state. However, for an accurate assessment of the chloride's influence, all the significant reactions with the chloride species should be added.

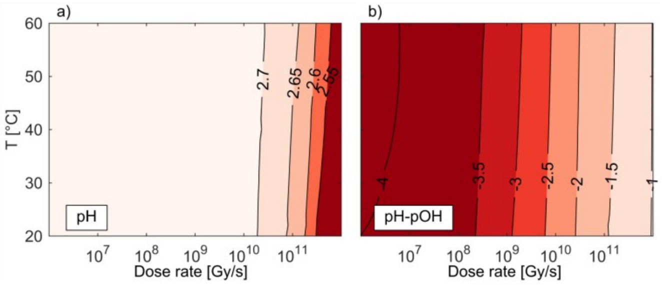

Figure S3. a) Equilibrium *pH* of the solution containing Au species. b) Equilibrium difference between *pH* and *pOH*. Initial *pH* = 2.8, maximum pressure 1 bar.

Calculation of pH and pOH: The Au ions can form hydroxide species and other aureate species at higher values of *pH* (>7) and at high redox potentials.[10] In order to evaluate the most stable gold species for the set experimental conditions the values of *pH* (Figure S3a) and *pH-pOH* (Figure S3b) were calculated. The graph indicates that the *pH* is stable at the initial *pH* value of 2.8 for the broad range of dose rates from 10^6 up to 10^{10} Gy/s. Above the indicated dose rate the *pH* value decreases from 2.7 to 2.55, when the dose rate is increased from 10^{10} to 10^{12} Gy/s, respectively. Relative to the dose rate, it was concluded that the pH variations are not significantly influenced by a change of the temperature in the liquid-cell TEM. However, to determine the chemical state of the gold species the OH^- concentration is also important. The difference *pH-pOH* is equal to $log_{10}([OH^-]/[H^+])$ and ranges between -4 and -1, mainly influenced by the changes in the dose rates. Due to the relatively low *pH* value (2.7-2.6) and the *pH-pOH* value between -4 and -1 (a more acidic solution) at all dose rates and temperatures, this study confirms that the dissolved Au species are in the form $[AuCl_2]^-$.

Table S3. Reaction-rate constants of the radiolysis products with gold species

Au spec.	Radiolysis spec.	$k_{20°C}$ [$M^{-1}s^{-1}$]	Source	E_A [kJ/mol]	Source	$A_{calculated}$
Au^+	e^-_{aq}	8.0×10^9	[11]	12.98[b]		1.64×10^{12}
	$H^•$	8.0×10^9 [a]	[11]	15.09[c]		3.91×10^{12}
	H_2O_2	0 [j]	[12]	/	[12]	0
	HO_2^-	1.89[d]	[12]	25.8 [d,e]	[12]	7.46×10^4
	H_2	7.4×10^{-3} [d, f]	[13]	94.1[d,g]	[14]	4.28×10^{14}
	$HO_2^•$	1.89 [d,h]		25.8 [d,h]		7.46×10^4
	$O_2^{•-}$	1.89 [d,h]		25.8 [d,h]		7.46×10^4
Au^0	$OH^•$	1.83×10^9[i]		13.0 [i]		3.80×10^{11}

Please do not adjust margins

[a] Assumed the same k_{20} °C, as for the reaction between e^-_{aq} and Au^+, because the reaction rate constants oGf : Au^{+3}+$H^•$/ e^-_{aq} →Au^{+2}+H^+/H_2O are similar ((5.7 +/- 1.5) × 10^9 $M^{-1}s^{-1}$ [11]).

[b] E_A assumed the same as for the reaction e^-_{aq} +$O_2^{•-}$(+ H_2O)→HO_2^- +OH^-, because both reactions are diffusion controlled and $O_2^{•-}$ as the $[AuCl_2]^-$ negatively charged.

[c] E_A assumed the same as for the reaction $H^•$ + $O_2^{•-}$→ HO_2^-, because both reactions are diffusion controlled and $O_2^{•-}$ is as the $[AuCl_2]^-$ negatively charged.

[d] Rate for the presence of a gold catalyst, as given in the literature.

[e] E_A from the overall observed constant is used (apparent activation energy).[12]

[f] Rate expression $k[Au^o][H_2]$, at $pH = 7$ [13].

[g] E_A assumed for the H_2 dissociation on Au (211) [14], since is the dissociation step .

[h] Assumed the same rate and activation energy as for the reaction HO_2^-+Au^+→$HO_2^•$+Au^0. $HO_2^•$ and $O_2^{•-}$ are thermodynamically much stronger reducing agents (E_{NERNST} = -0.27 and 0.05 respectively (Table 1)) than HO_2^- (E_{NERNST} = 0.65 (Table 1)). However, in the DFT study on Au (111) [15] they show, that the reaction H_2O_2*→HO_2*+H* has a lower activation energy (E_A = 113 kJ/mol, ΔE = 106 kJ/mol) than the reaction HO_2*→ O_2*+H* (E_a = 141 kJ/mol, ΔE = 85 kJ/mol). Also, it is found that at higher *pH* (10-13) the reduction of $HAuCl_4$ with H_2O_2 is more significant and much faster than at neutral *pH* (7–8).[12] This can be due to a larger amount of HO_2^- and $O_2^•$. The electron-transfer reactions are generally fast and it is expected that $O_2^•$ will reduce the Au species with a rate similar to $H^•$ or e^-_{aq}. However, in the case of low pH, $O_2^•$ is protonated to $HO_2^•$, which needs to be broken on the Au surface to form O_2 and H*, and this is a much slower process, as seen from the DFT calculations.[15] Because of the lack of experimental data on the kinetics of $HO_2^•$ and $O_2^{•-}$ with Au^+ we assume the same rate as for the case of the reaction of HO_2^- with Au^+. However, for the experiments at high *pH,* where the amount of HO_2^- and $O_2^•$ is significant, corrected reaction rates should be used.

[i] Assumed the same rate coefficient as for the reaction of Au^++$OH^•$→Au^{+2} +OH^-. The rates of dissolution of Au^0 with a strong oxidizing or complexing agent without stirring are usually diffusion controlled.[16] Because of zero stirring, the reaction is diffusion controlled. For the case of the dissolution of Au in a cyanide solution the activation energies are 12–21 kJ/mol.[16] The temperature dependence of the diffusivity constant for water self-diffusion, which is similar to the $OH^•$ diffusion constant, is used to calculate the activation energy.[1]

[j] The value is 0 because the mechanism of Au ion reduction proceeds through HO_2^-.[12] In the kinetic model we have already included the reversible reaction between H_2O_2 and HO_2^-, and therefore the contribution to the reduction of the H_2O_2 is 0.

1.3 Explanation of redox trends due to temperature change:

The influence of the temperature on $[Au^o]/[Au^+]$ increases with increasing pressure (Figure 3), which points to the influence of the H_2 and O_2 solubility in the liquid phase. The temperature dependence of the O_2 solubility is significantly higher when compared with the solubility of H_2 in water (Table S4).

Table S4. Solubility of the H_2 and O_2 gases in water.

T	$\frac{1}{K_H}\Big\vert_{O_2}\left[\frac{mol}{L\ bar}\right]$	$\frac{1}{K_H}\Big\vert_{H_2}\left[\frac{mol}{L\ bar}\right]$
20 °C	1.40×10^{-3}	8.08 ×10^{-4}
60 °C	0.88×10^{-3} (decrease by 37% relative to 20 °C)	7.28 ×10^{-4} (decrease by 9.9% relative to 20 °C)

To prove the impact of the solubility's temperature dependence we performed a sensitivity analysis for the temperature-dependent part of the solubility relation (of both gases at the same time). We calculated the Au redox ratios for the different *factor* values: 0.5, 1 and 2.

$$H_i(T(K)) = exp(-A + \frac{B}{factor(T - 293.15\ K) + 293} \qquad (S4)$$

Table S5. Parameters of the temperature-dependent Henry constant. [17,18]

Coef.	H_2	O_2
A	48.1611	66.7354
B	5528.45	8747.55
C	16.8893	24.4526

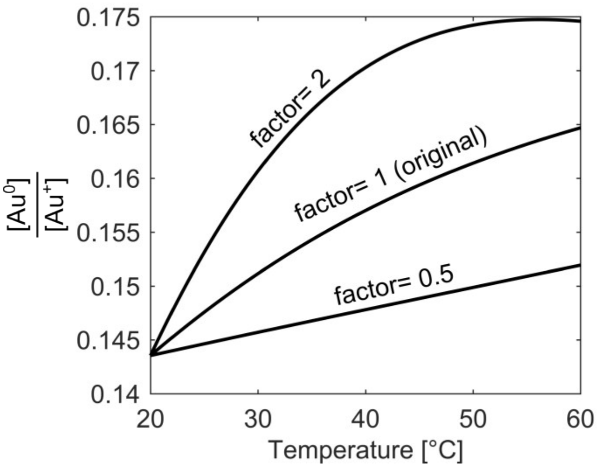

Figure S4. Temperature dependence of the Au redox ratio at different sensitivity factors of the H_2 and O_2 solubilities. Dose rate 10^9 Gy/s, total concentration of gold species is 1.5 mM with an initial pH 2.8, and pressure 5 bar.

From Figure S4 we can see that the temperature dependence of the solubility influences the Au redox ratio. When we increase the temperature dependence of the gases' solubility (*factor* = 2) the

This journal is © The Royal Society of Chemistry 20xx

Please do not adjust margins

Please do not adjust margins

redox ratio increases faster with temperature, and when we decrease the temperature dependence of the gases' solubility (*factor* = 0.5) the redox ratio increases more slowly. The concentration of O_2 in the liquid phase decreases much faster than the concentration of H_2. Because H_2 is a strong $OH^\bullet$ radical scavenger ($k_{20\,°C}$ = 3.4×10^{7} $M^{-1}s^{-1}$) and O_2 is a strong scavenger of $H^\bullet$ and e^-_{aq} ($k_{20\,°C}$ = 1.2×10^{10}, 2.1×10^{10} $M^{-1}s^{-1}$, respectively), the ratio $\frac{[H^\bullet]+[e^-_{aq}]}{[OH^\bullet]}$ (consequently Au redox ratio), depends on the concentrations of O_2 and H_2.

2. MODEL VERIFICATION BY LOW-DOSE EXPERIMENTS USING γ RADIATION

The conditions used for our model verification were collected from several sources [6,19,20] for the bulk water radiolysis in solutions of H_2O_2, O_2, and H_2 by using *γ* rays as a source of radiation, which is similar to electron radiation low-LET (linear energy transfer). The experiments were performed at different dose rates and solute concentrations.

Table S6. Detailed conditions of the radiolysis model's verification.

source	Fig.	Line	Dose rate [Gy/s]	*T* [°C]	c_{init} [M]			
					pH_{init}	H_2O_2	O_2	H_2
Pastina, LaVerne 2001 [6]	4a	1	0.250	25	7	4.88×10^{-5}	0	0
		2	0.250	25	7	4.88×10^{-3}	0	8.00×10^{-6}
		3	0.250	25	7	4.88×10^{-5}	0	8.00×10^{-5}
		4	0.250	25	7	4.88×10^{-5}	0	8.00×10^{-4}
Hayon 1964 [19]	4b	1	0.205	25	7	1.20×10^{-3}	0	0
		2	0.205	25	7	7.57×10^{-4}	0	0
		3	0.205	25	7	5.35×10^{-4}	0	0
		4	0.205	25	7	3.20×10^{-4}	0	0
		4	0.205	25	7	1.39×10^{-4}	0	0
Hochandel 1952 [20]	4c	1	0.653	25	7	0	3.32×10^{-4}	4.90×10^{-4}
		2	0.653	25	7	0	7.50×10^{-5}	7.31×10^{-4}
		3	0.653	25	7	0	1.25×10^{-3}	0

3. MODEL VERIFICATION BASED ON Au PRECIPITATION/DISSOLUTION LCTEM EXPERIMENTS

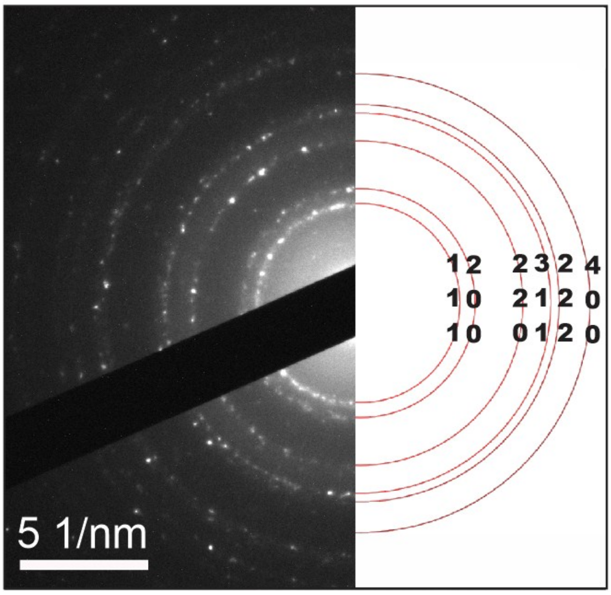

Figure S5. Ex-situ SAED analysis of NPs formed during in-situ LCTEM experiment, indicating the FCC structure of gold.

4. GRAPHICAL ILLUSTRATION OF THE AU PRECIPITATION/DISSOLUTION CONTROLLED BY THE RADICAL-INDUCED REDOX CHEMISTRY INSIDE THE LIQUID-CELL TEM

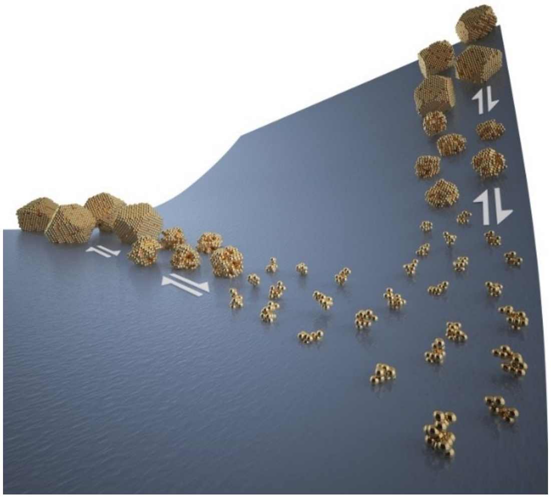

Figure S6. Growth and dissolution of Au particles are reversible and can be controlled by different operating conditions in the liquid-cell TEM. The three-dimensional surface represents the value of the equilibrium [Au°]/[Au+] ratio. For example, high-slope regions signify higher gradients in [Au°]/[Au+], hence the gold's tendency for precipitation. The vertical component is related to the increase of the dose rate, while the horizontal component indicates a temperature increase.

Please do not adjust margins

Please do not adjust margins

NOTES AND REFERENCES

(S1) Elliot, A.J. Bartels, D. M.; Elliot. The Reaction Set, Rate Constants and g-Values for the Simulation of the Radiolysis of Light Water over the Range 20° to 350°C Based on Information Available in 2008; Report AECL No. 153-127160-450-001; Atomic Energy of Canada Ltd.:Mississauga, Ontario, Ca. **2009**.

(S2) Schneider, N. M.; Norton, M. M.; Mendel, B. J.; Grogan, J. M.; Ross, F. M.; Bau, H. H.; Frances, M.; Bau, H. H. Electron-Water Interactions and Implications for Liquid Cell Electron Microscopy. *J. Phys. Chem. C* **2014**, *118*, 22373–22382.

(S3) Green, N. J. B.; Pilling, M. J.; Pimblott, S. M.; Clifford, P. Stochastic Modeling of Fast Kinetics in a Radiation Track. *J. Phys. Chem.* **1990**, *94*, 251–258.

(S4) Anderson, R. F.; Vojnovic, B.; Michael, B. D. The Radiation-Chemical Yields of H_3O^+ and OH^- as Determined by Nanosecond Conductimetric Measurements. *Radiat. Phys. Chem.* **1985**, *26*, 301–303.

(S5) Hill, M. A.; Smith, F. A. Calculation of Initial and Primary Yields in the Radiolysis of Water. *Radiat. Phys. Chem.* **1994**, *43*, 265–280.

(S6) Pastina, B.; Laverne, J. A. Effect of Molecular Hydrogen on Hydrogen Peroxide in Water Radiolysis. *J. Phys. Chem. A* **2001**, *105*, 9316–9322.

(S7) Park, J. H.; Schneider, N. M.; Grogan, J. M.; Reuter, M. C.; Bau, H. H.; Kodambaka, S.; Ross, F. M. Control of Electron Beam-Induced Au Nanocrystal Growth Kinetics through Solution Chemistry. *Nano Lett.* **2015**, *15*, 5314–5320.

(S8) Atinault, E.; De Waele, V.; Schmidhammer, U.; Fattahi, M.; Mostafavi, M. Scavenging of Es^- and OH⍰ Radicals in Concentrated HCl and NaCl Aqueous Solutions. *Chem. Phys. Lett.* **2008**, *460*, 461–465.

(S9) Armstrong, D. A., Huie; R. E.; Lymar, S.;Koppenol, W. H.;Merényi, G.; Neta, P.; Ruscic, B.; Stanbury, D. M.; Steenken, S. Standard Electrode Potentials Involving Radicals in Aqueous Solution : Inorganic Radicals (IUPAC Technical Report). *Pure Appl. Chem.* **2015**, *87*, 1139–1150.

(S10) Kaye, M. H.;Thompson, W. T. Pourbaix Diagrams for Multielement Systems. In *Uhlig's corrosion handbook, Third Edition*; Revie, W. R., Ed.; John Wiley & Sons, Inc.: Hoboken, New Jersey, 2011; pp 103–122.

(S11) Ghosh Mazumdar, A. S. and Hart, E. J. A Pulse Radiolysis Study of Bivalent and Zerovalent Gold in Aqueous Solutions. In *Radiation Chemistry*; Hart, E. J., Ed.; American Chemical Society, 1968; pp 193–209.

(S12) Pacławski, K. and Fitzner, K. Kinetics of Reduction of Gold(III) Complexes Using H_2O_2. *Metall. Mater. Trans. B* **2006**, *37B*, 703–714.

(S13) Ershov, B. G.; Abkhalimov, E. V.; Solovov, R. D.; Roldughin, V. I. Gold Nanoparticles in Aqueous Solutions: Influence of Size and PH on Hydrogen Dissociative Adsorption and Au(III) Ion Reduction. *Phys. Chem. Chem. Phys.* **2016**, *18*, 13459–13466.

(S14) Wang, S.; Petzold, V.; Tripkovic, V.; Kleis, J.; Howalt, J. G.; Skúlason, E.; Fernández, E. M.; Hvolbæk, B.; Jones, G.; Toftelund, A.; Falsig, H.; Björketun, M.; Studt, F.; Abild-Pedersen, F.; J., R.; Nørskov, J. K.; Bligaard, T. Universal Transition State Scaling Relations for (de)Hydrogenation over Transition Metals. *Phys. Chem. Chem. Phys.* **2011**, *13*, 20760–20765.

(S15) Yang, Y.; Dai, C.; Fisher, A.; Shen, Y. A Full Understanding of Oxygen Reduction Reaction Mechanism on Au(111) Surface. *J. Phys. Condens. Matter* **2017**, *29*, 365201–365208.

(S16) Habahchi, F. *Kinetics and Mechanism of Gold and Silver Dissolution in Cyanide Solution*; Montana Bureau of Mines Geological Bulletin 59, 1967.

(S17) Young, C. L. Volume 5/6, Hydrogen and Deuterium. In *IUPAC-NIST Solubility Database*; Pergamon Press: Oxford, U.K., 1981; pp 1–3.

(S18) Battino, R. Volume 7, Oxygen and Ozone. In *IUPAC-NIST Solubility Database*; Pergamon Press: Oxford, U.K., 1981; pp 1–5.

(S19) Hayon, E. Radiolysis of Air-Free Aqueous Solutions of Hydrogen Peroxide. *Trans. Faraday Soc.* **1964**, *60*, 1059–1067.

(S20) Hochanadel, C. J. Effects of Cobalt γ-Radiation on Water and Aqueous Solutions. *J. Phys. Chem.* **1952**, *56*, 587–594.

This journal is © The Royal Society of Chemistry 20xx

Please do not adjust margins
